# Two polymorphs of *trans*-[3-(3-nitro­phen­yl)oxiran-2-yl](phen­yl)methanone

**DOI:** 10.1107/S2056989016010239

**Published:** 2016-06-28

**Authors:** Fred H. Greenberg, Alexander Y. Nazarenko

**Affiliations:** a Chemistry Department, SUNY Buffalo State, 1300 Elmwood Ave, Buffalo, NY 14222, USA

**Keywords:** crystal structure, chalcone oxide, polymorphism

## Abstract

The title compound, C_15_H_11_NO_4_, crystallizes in two polymorphic forms, centrosymmetric monoclinic and chiral ortho­rhom­bic. The geometry of the mol­ecules in the two polymorphs is slightly different, possibly due to inter­molecular inter­actions. There are no classical hydrogen bonding in these two structures. However, a number of C—H⋯O inter­molecular inter­actions, involving both O atoms of the nitro as well the benzoyl groups, stabilize the crystal structures.

## Abstract 2

The title compound, C_15_H_11_NO_4_, crystallizes in two polymorphic forms, centrosymmetric monoclinic and chiral ortho­rhom­bic. The geometry of the mol­ecules in the two polymorphs is slightly different, possibly due to inter­molecular inter­actions. A number of C—H⋯O inter­molecular inter­actions, involving both O atoms of the nitro as well the benzoyl groups, stabilize the crystal structures.

## Chemical context   

The title compound is a substituted chalcone oxide, a representative of a large group of organic compounds which are precursors for pharmaceutically significant flavonoids (Marais *et al.*, 2005 ▸). As for most biologically important mol­ecules, chirality plays an important role in their reactions. These compounds can be also considered as isomers of substituted di­benzoyl­methanes.
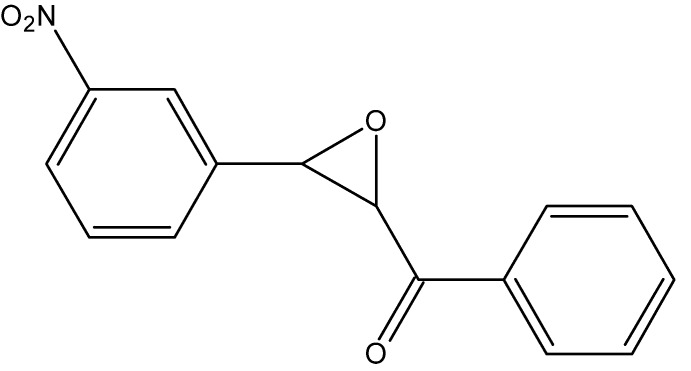


The simplest compound of this group, 1,3-diphenyl-2,3-epoxy­propan-1-one (2-benzoyl-3-phenyl­oxirane, benzal­aceto­phenone oxide) was isolated by Widman (1916 ▸) using Darzens condensation of benzaldehyde and bromo­aceto­phenone in the presence of sodium ethoxide. When *m*-nitro­benzaldehyde was employed in this reaction, the title compound was obtained (Bodforss, 1916 ▸). The original publication mentioned the possibility of two different types of colorless crystals, both having the same melting point of 391 K. Later, a number of alternative synthetic routes were developed, including Claisen condensation of *m*-nitro­benzaldehyde with aceto­phenone with subsequent oxidation (Roth & Schwarz, 1961 ▸). The authors described the title compound as pale-yellow needles. A one-pot version of this synthesis was reported recently (Ngo *et al.*, 2014 ▸). Preparation of nitro­chalcone oxides seems to be one of the simplest condensation reactions and therefore attractive for use in undergraduate organic chemistry teaching laboratories. The inter­esting observation of possible polymorphism in the original publication encouraged us to conduct a structural study, exactly one hundred years after the first preparation of this compound had been reported.

## Structural commentary   

**Figure 1:**
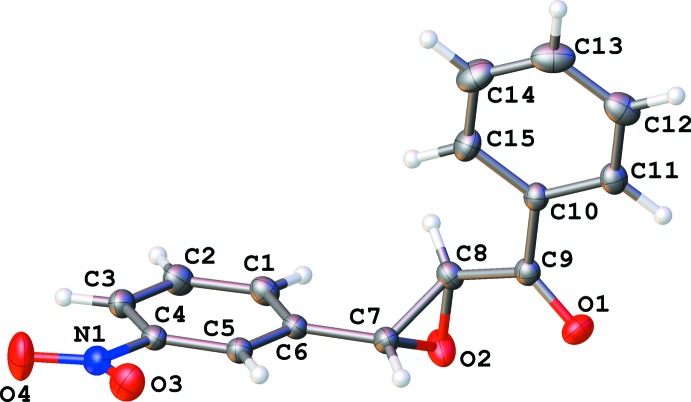
Numbering scheme of the title compound with 50% probability ellipsoids (monoclinic polymorph).

**Figure 3:**
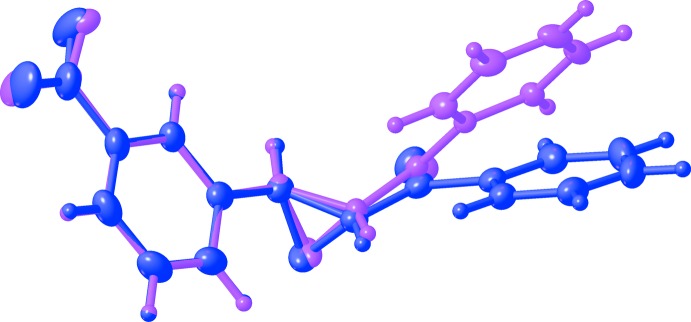
Overlay of the two polymorphic mol­ecules (nitro­phenyl group matching atoms). Pink: monoclinic (after inversion); purple: ortho­rhom­bic.

The title compound, C_15_H_11_NO_4_, crystallizes in two polymorphic forms, centrosymmetric monoclinic (1) and chiral ortho­rhom­bic (2). Bond lengths and angles in the mol­ecules of both polymorphs are very similar (Figs. 1 ▸ and 2 ▸). However, a mol­ecular overlay (Fig. 3 ▸) reveals some difference in conformation, possibly because of different types of inter­molecular inter­actions.

All atoms of the title polymorphs, except the oxiran ring hydrogen atoms, are located close to one of three planes: the benzene ring mean plane of the nitro-phenyl group (*A*), the oxiran ring plane (*B*), and the benzene ring plane of the benzoyl group (*C*). The largest deviations from these planes are −0.2003 (14) and 0.0457 (15) for O3 and O4 (monoclinic polymorph), 0.091 (4) and −0.189 (3) for O3 and O4 (ortho­rhom­bic), and 0.3398 (14) and 0.065 (3) for atom O1 in the monoclinic and the ortho­rhom­bic forms, respectively. Planes *A* and *B* are almost perpendicular in both polymorphs (Table 1 ▸). The angles between the two other planes differ significantly (Table 1 ▸).

## Supra­molecular features   

There are no classical hydrogen bonds in these two polymorphs. In the mol­ecules, areas of negative electrostatic potential are located in the vicinity of all four oxygen atoms. Areas near hydrogen atoms are obviously positive, providing a tool for inter­molecular inter­actions. This expectation is supported by the packing data. In both polymorphs, the two oxygen atoms O3 and O4 of the nitro group and oxygen atom O1 of the carbonyl group act as acceptors for C—H⋯O hydrogen bonds. Despite being relatively weak, such bonds play a significant role in inter­molecular inter­actions (Desiraju & Steiner, 1999 ▸). Hydrogen atom H5 of the nitro­phenyl group makes short contacts with the O1 oxygen of the carbonyl group in both cases. However, the short contacts involving the nitro group oxygen atoms O3 and O4 (Tables 2 ▸ and 3 ▸) are different in the two polymorphs. In the ortho­rhom­bic polymorph, the oxiran ring hydrogen atom H8 makes a short contact to one of the nitro group oxygen atoms. Another oxiran ring hydrogen atom makes a contact with carbonyl group oxygen O1 that is slightly longer than usual for C—H⋯O bonding [*D*—H 1.000 (19), H⋯*A* 2.64 (2), *D*⋯*A* 3.419 (2) Å; *D–*-H⋯A 134.7 (2)°]. There are a number of C—H⋯π contacts that are on the long side of what is still considered to be an attractive inter­action: C12—H12⋯C14(*x*, 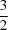 − *z*, 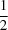 + *z*) and C1—H1⋯C13(1 − *x*, 1 − *y*, 2 − *z*) in the monoclinic form with C⋯C distances of 3.7870 (15) and 3.7637 (12) Å, respectively, and C14—H14⋯C2(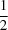 − *x*, 1 − *y*, −0.5 + *z*) in the ortho­rhom­bic form with a C⋯C distance of 3.731 (3) Å.

Without strong inter­molecular bonding, the close-packing principle directs the assembly of mol­ecules in the crystal. A multi-step approach to assembling is sometimes referred as the Kitaigorodskii Aufbau Principle (KAP) and may consist of the following sequence (Kitaigorodskii, 1961 ▸; Perlstein, 1994 ▸): (*a*) a single mol­ecule or a number of mol­ecules forming a unit; (*b*) units join up to form a chain; (*c*) chains assemble to make a 2D surface and (*d*) surfaces are stacked to form a crystal.

**Figure 4:**
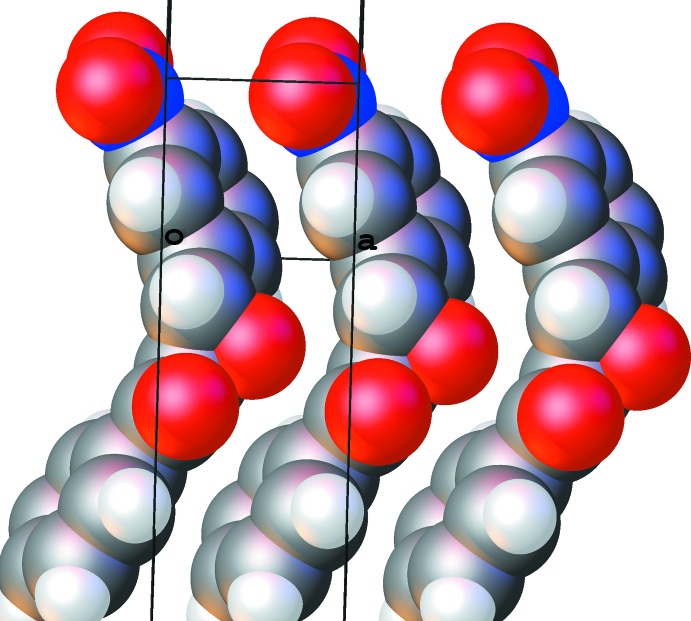
Packing diagram for the ortho­rhom­bic polymorph. One chain of mol­ecules along the [100] axis is shown.

**Figure 5:**
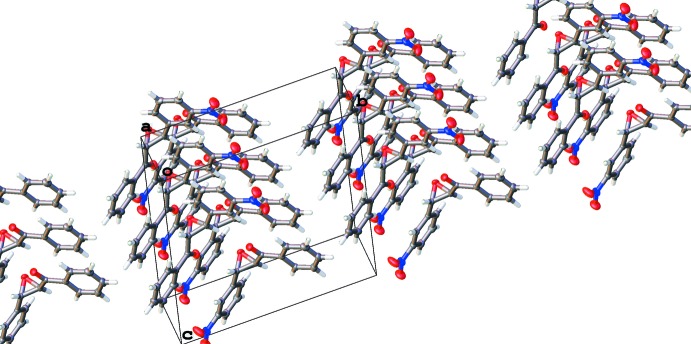
Packing diagram for the ortho­rhom­bic polymorph. Chains of mol­ecules with one direction are stacked in the (001) plane.

**Figure 6:**
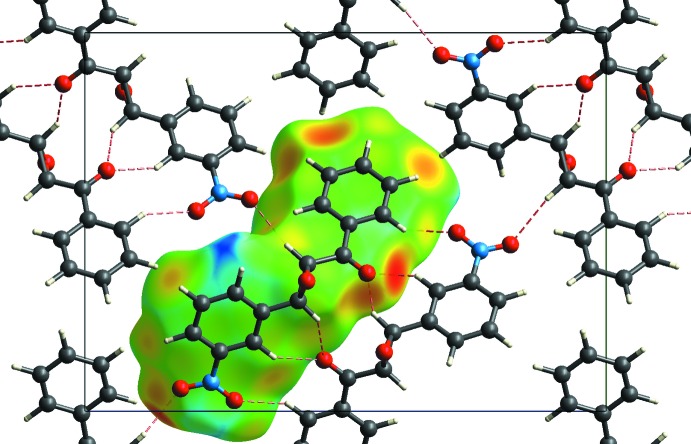
Packing diagram for the ortho­rhom­bic polymorph. View along the *a* axis. Hirshfeld surface shown for one mol­ecule (calculated using *CrystalExplorer;* Wolff *et al.*, 2012 ▸).

This sequence can be traced in the structure of the ortho­rhom­bic polymorph. Mol­ecules of the title compound are stacked to form a chain along [100] axis (Fig. 4 ▸). An oxiran group forms a ‘wedge’ that fits into a concave ‘pocket’ between two phenyl rings of the next mol­ecule. The inter­atomic distances between oxiran oxygen atom O2 and the corresponding carbon atoms are unusually short: O2⋯C7(1 + *x*, *y*, *z*) = 3.113 (2), O2⋯C8(1 + *x*, *y*, *z*) = 2.960 (2) and O2⋯C9(1 + *x*, *y*, *z*) = 2.979 (2) Å. Two separate causes can make these short contacts possible: (i) dipole–dipole attraction of consecutive oxiran groups and (ii) close packing of recurrent flat benzoyl and nitro­phenyl groups with the distances between their mean planes being 3.472 (2) and 3.493 (2), respectively. Because all these groups are parallel, there is no hydrogen bonding within the chain. At the next level, chains are packed in the (001) plane *via* a 2_1_ symmetry operation, with all oxiran groups oriented in one direction (Fig. 5 ▸). Finally, chains are closely packed with the next 2_1_ operation, forming a crystal with favorable hydrogen bonding (Fig. 6 ▸).

**Figure 7:**
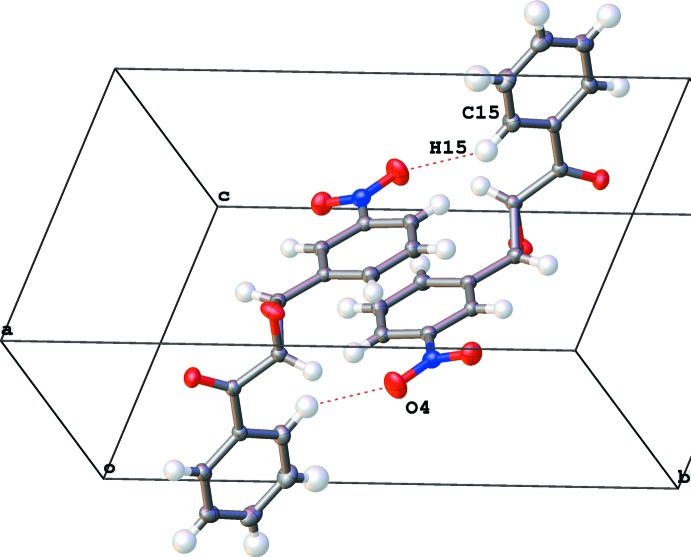
Packing of the monoclinic polymorph. Two mol­ecules are related by inversion.

**Figure 8:**
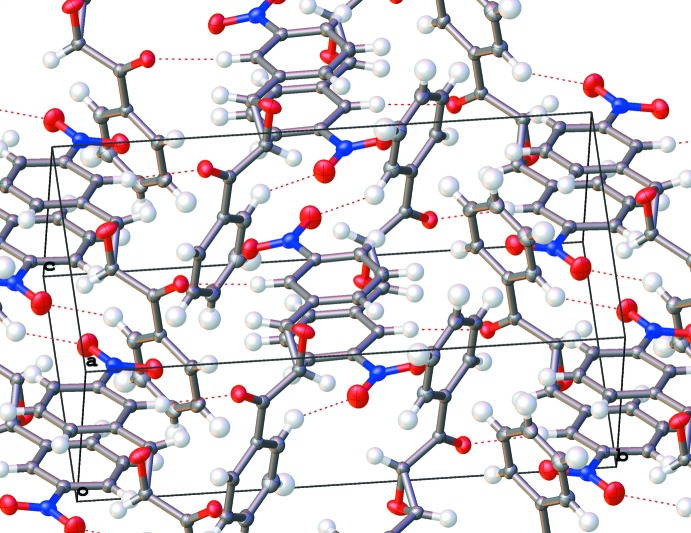
Packing diagram of the monoclinic polymorph. Mol­ecules are assembled in the (100) plane.

The monoclinic form of the title compound is possible only if the starting solution contains a racemic mixture. In the first step, two mol­ecules are π-stacked *via* inversion centers *via* their nitro­phenyl groups and two symmetric hydrogen bonds (Fig. 7 ▸). The distance between the parallel planes of these phenyl rings is 3.4115 (10), which is slightly longer than in polyaromatic hydro­carbons (3.38 Å; Kitaigorodskii, 1961 ▸) and indicates very close packing. These centrosymmetric units are assembled in the (100) plane *via* a system of hydrogen bonds (Fig. 8 ▸). The stacking planar assemblies in the 3D crystal uses no additional hydrogen bonding.

The assembling sequence is mechanically more straightforward in the case of the chiral ortho­rhom­bic form, which results in favorable formation of the ortho­rhom­bic polymorph. The absence of an enanti­omer requirement may also make it kinetically more favorable. These two factors can serve as a qualitative explanation of the preferred formation of the ortho­rhom­bic form upon crystallization from alcohols or from hexane. The monoclinic form has a slightly smaller cell volume (see Table 4 ▸) and, therefore, closer packing of mol­ecules, an indication that the monoclinic form might be the thermodynamically slightly more stable of the polymorphs according to Burger and Ramberger’s Density Rule (Burger & Ramberger, 1979*a* ▸,*b* ▸). Nevertheless, the packing of the two forms is significantly different and transition from one form to another requires dissolution of the crystal. This observation explains the kinetic stability of both forms at room temperature and at 173 K.

## Database survey   

There are sixteen reported chalcone oxide structures deposited in the Cambridge Structural Database (CSD Version 5.37; Groom *et al.*, 2016 ▸). Of these structures, six report hy­droxy- and meth­oxy-substituted mol­ecules with strong inter­molecular inter­actions. The closest to our study is [3-(4-nitro­phen­yl)oxiran-2-yl](phen­yl)methanone (refcode COVKAB; Obregón-Mendoza *et al.*, 2014 ▸). In this case, the oxiran oxygen atom makes short contacts instead of the benzoyl group; the *p*-nitro­phenyl ring is practically flat. The simplest unsubstituted chalcone oxide was recently reported (refcode TIBXIM; Zaidi *et al.*, 2007 ▸). In this structure, like in our case, only the benzoyl group oxygen atom makes short inter­molecular contacts. Chains similar to those in the ortho­rhom­bic form of the title mol­ecule are present in the chiral *P*2_1_ crystal of [3-(4-chloro­phen­yl)oxiran-2-yl](phen­yl)methanone (refcode QECFAF; Bakó *et al.*, 1999 ▸). However, the distances between oxiran oxygen atom and the subsequent carbon atoms are much longer than in the present case.

## Synthesis and crystallization   

**Figure 9:**
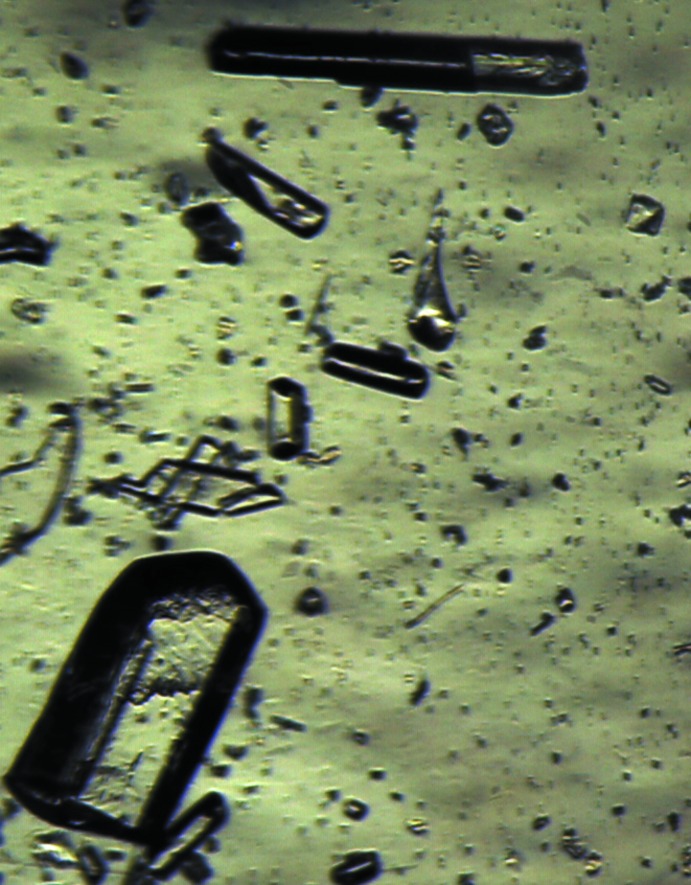
Crystals of different polymorphs in solution. Large blocks are monoclinic, needles are ortho­rhom­bic.

The title compound was obtained *via* the classic route (Bodforss, 1916 ▸). Mass-spectrum (EI): 269 (*M*+, 20%), 105 (PhCO+, 100), 77 (Ph+, 60). Because all precursor compounds were non-chiral and synthetic conditions should not induce chirality, we expected to see a racemic product. Crystallization from hexane yielded colorless thin needles suitable for single-crystal investigation. X-ray diffraction data revealed the chiral ortho­rhom­bic space group *P*2_1_2_1_2_1_. Crystallization from ethanol produced better quality crystals of the same polymorph, one of which was used in this study. After two weeks of standing at 273 K, a number of large (up to 1 mm) crystals were observed in the remaining ethanol solution (Fig. 9 ▸). A suitable crystal was cut to dimensions appropriate for X-ray analysis. It turned out to be a monoclinic *P*2_1_/*c* polymorph of the same compound. Several crystals of different shape, also formed from the same solution, resulted to be of a benzoin admixture.

## Refinement   

Crystal data, data collection and structure refinement details are summarized in Table 4 ▸. The structure of the ortho­rhom­bic polymorph was refined as a two-component inversion twin. All hydrogen atoms in the monoclinic form were refined in isotropic approximation. In the ortho­rhom­bic form, the oxiran ring hydrogen atoms H7 and H8 were refined in isotropic approximation with *U*_iso_ = 1.2*U*_iso_(C). All aromatic hydrogen atoms in this mol­ecule were refined with riding coordinates and *U*_iso_ = 1.2*U*_iso_(C).

For the monoclinic polymorph structure, positive residual density was observed at all bonds between non-hydrogen atoms, demonstrating the limitations of the atom-in-mol­ecule approach for high-resolution structures of organic mol­ecules.

## Supplementary Material

Crystal structure: contains datablock(s) 1, 2. DOI: 10.1107/S2056989016010239/zl2667sup1.cif

Structure factors: contains datablock(s) 1. DOI: 10.1107/S2056989016010239/zl26671sup5.hkl

Supporting information file. DOI: 10.1107/S2056989016010239/zl26671sup7.cdx

Structure factors: contains datablock(s) 2. DOI: 10.1107/S2056989016010239/zl26672sup6.hkl

Supporting information file. DOI: 10.1107/S2056989016010239/zl26671sup5.cml

Supporting information file. DOI: 10.1107/S2056989016010239/zl26672sup6.cml

CCDC references: 1487266, 1487265

Additional supporting information:  crystallographic information; 3D view; checkCIF report

## Figures and Tables

**Figure 2:**
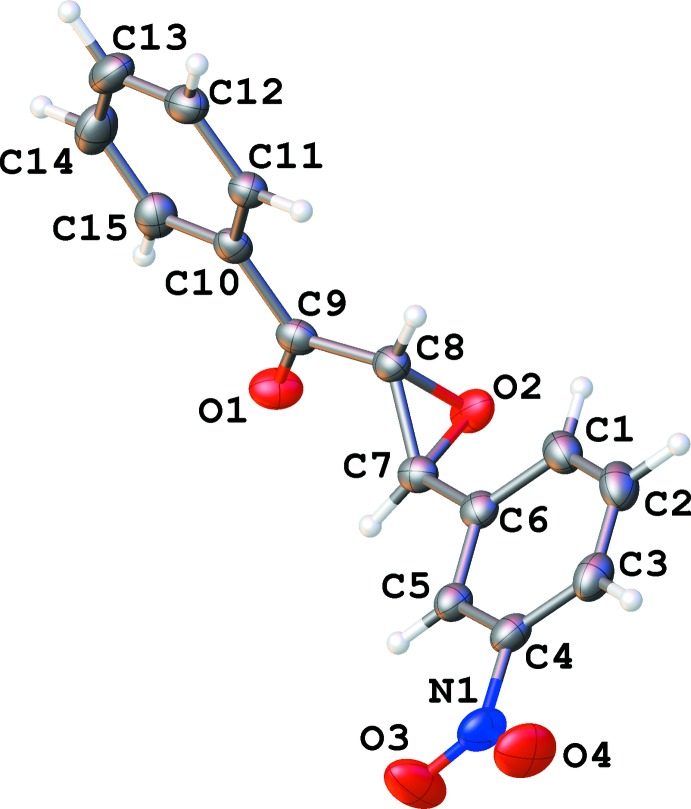
Numbering scheme of the title compound with 50% probability ellipsoids (ortho­rhom­bic polymorph).

**Table 1:** Angles between planes (°) Plane *A*: mean plane of the *m*-nitro­phenyl benzene ring; plane *B*: oxirane ring; plane *C*: mean plane of the benzoyl benzene ring.

Planes	monoclinic polymorph	ortho­rhom­bic polymorph
*A*/*B*	99.78 (3)	97.97 (10)
*A*/*C*	102.36 (3)	66.21 (6)
*B*/*C*	55.53 (5)	75.54 (10)

**Table 2:** Hydrogen-bond geometry (Å, °) for the monoclinic polymorph[Chem scheme1]

*D*—H⋯*A*	*D*—H	H⋯*A*	*D*⋯*A*	*D*—H⋯*A*
C5—H5⋯O1^i^	0.962 (13)	2.335 (13)	3.2791 (11)	167.0 (11)
C13—H13⋯O3^ii^	0.945 (17)	2.490 (17)	3.3530 (13)	152.0 (13)
C15—H15⋯O4^iii^	0.992 (14)	2.381 (15)	3.2426 (13)	144.8 (12)

Symmetry codes: (i) 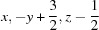; (ii) 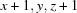; (iii) 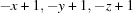.

**Table 3:** Hydrogen-bond geometry (Å, °) for the orthorhombic polymorph[Chem scheme1]

*D*—H⋯*A*	*D*—H	H⋯*A*	*D*⋯*A*	*D*—H⋯*A*
C5—H5⋯O1^i^	0.94 (1)	2.35 (1)	3.209 (2)	151 (1)
C8—H8⋯O4^ii^	0.96 (2)	2.49 (2)	3.401 (2)	158 (2)
C15—H15⋯O3^iii^	1.00 (1)	2.51 (2)	3.411 (2)	150 (1)

Symmetry codes: (i) 
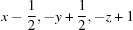
; (ii) 
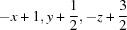
; (iii) 
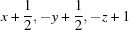
.

**Table 4:** Experimental details

	monoclinic polymorph	orthorhombic polymorph
Crystal data		
Chemical formula	C_15_H_11_NO_4_	C_15_H_11_NO_4_
*M*_r_	269.25	269.25
Crystal system, space group	Monoclinic, *P*2_1_/*c*	Orthorhombic, *P*2_1_2_1_2_1_
Temperature (K)	173	173
*a*, *b*, *c* (Å)	7.8463 (5), 16.2514 (9), 10.2032 (6)	4.1615 (2), 14.7498 (6), 20.3168 (8)
α, β, γ (°)	90, 108.839 (2), 90	90, 90, 90
*V* (Å^3^)	1231.35 (13)	1247.07 (9)
*Z*	4	4
Radiation type	Mo *K*α	Cu *K*α
μ (mm^−1^)	0.11	0.88
Crystal size (mm)	0.5 × 0.45 × 0.4	0.44 × 0.07 × 0.06

Data collection		
Diffractometer	Bruker PHOTON-100 CMOS	Bruker PHOTON-100 CMOS
Absorption correction	Multi-scan (*SADABS*; Bruker, 2014 ▸)	Multi-scan (*SADABS*; Bruker, 2014 ▸)
*T*_min_, *T*_max_	0.942, 0.969	0.798, 0.950
No. of measured, independent and observed [*I* > 2σ(*I*)] reflections	60976, 5593, 4604	39306, 2640, 2435
*R*_int_	0.031	0.037
(sin θ/λ)_max_ (Å^−1^)	0.820	0.636

Refinement		
*R*[*F* ^2^ > 2σ(*F* ^2^)], *wR*(*F* ^2^), *S*	0.044, 0.125, 1.06	0.029, 0.071, 1.06
No. of reflections	5593	2640
No. of parameters	225	197
H-atom treatment	All H-atom parameters refined	H atoms treated by a mixture of independent and constrained refinement
Δρ_max_, Δρ_min_ (e Å^−3^)	0.49, −0.23	0.13, −0.15
Absolute structure	–	Refined as an inversion twin
Absolute structure parameter	–	0.3 (2)

Computer programs: *APEX2* and *SAINT* (Bruker, 2013 ▸), *SHELXT* (Sheldrick, 2015*a* ▸), *SHELXL2014* (Sheldrick, 2015*b* ▸), *OLEX2* (Dolomanov *et al.*, 2009 ▸) and *CrystalExplorer* (Spackman & Jayatilaka, 2009 ▸).

## supplementary crystallographic information

### (1) [3-(3-Nitrophenyl)oxiran-2-yl](phenyl)methanone Crystal data

**Table:**

C_15_H_11_NO_4_	*F*(000) = 560
*M**_r_* = 269.25	*D*_x_ = 1.452 Mg m^−^^3^
Monoclinic, *P*2_1_/*c*	Mo *K*α radiation, λ = 0.71073 Å
*a* = 7.8463 (5) Å	Cell parameters from 9972 reflections
*b* = 16.2514 (9) Å	θ = 3.0–30.6°
*c* = 10.2032 (6) Å	µ = 0.11 mm^−^^1^
β = 108.839 (2)°	*T* = 173 K
*V* = 1231.35 (13) Å^3^	Block, colourless
*Z* = 4	0.5 × 0.45 × 0.4 mm

### (1) [3-(3-Nitrophenyl)oxiran-2-yl](phenyl)methanone Data collection

**Table:**

Bruker PHOTON-100 CMOS diffractometer	4604 reflections with *I* > 2σ(*I*)
Radiation source: sealedtube	*R*_int_ = 0.031
φ and ω scans	θ_max_ = 35.6°, θ_min_ = 3.0°
Absorption correction: multi-scan (SADABS; Bruker, 2014)	*h* = −11→12
*T*_min_ = 0.942, *T*_max_ = 0.969	*k* = −26→25
60976 measured reflections	*l* = −16→14
5593 independent reflections	

### (1) [3-(3-Nitrophenyl)oxiran-2-yl](phenyl)methanone Refinement

**Table:**

Refinement on *F*^2^	0 restraints
Least-squares matrix: full	Hydrogen site location: difference Fourier map
*R*[*F*^2^ > 2σ(*F*^2^)] = 0.044	All H-atom parameters refined
*wR*(*F*^2^) = 0.125	*w* = 1/[σ^2^(*F*_o_^2^) + (0.0672*P*)^2^ + 0.211*P*] where *P* = (*F*_o_^2^ + 2*F*_c_^2^)/3
*S* = 1.06	(Δ/σ)_max_ = 0.001
5593 reflections	Δρ_max_ = 0.49 e Å^−^^3^
225 parameters	Δρ_min_ = −0.23 e Å^−^^3^

### (1) [3-(3-Nitrophenyl)oxiran-2-yl](phenyl)methanone Special details

**Table:**

Experimental. SADABS-2014/5 (Bruker,2014/5) was used for absorption correction. wR2(int) was 0.0614 before and 0.0562 after correction. The Ratio of minimum to maximum transmission is 0.8403. The λ/2 correction factor is 0.00150.
Geometry. All esds (except the esd in the dihedral angle between two l.s. planes) are estimated using the full covariance matrix. The cell esds are taken into account individually in the estimation of esds in distances, angles and torsion angles; correlations between esds in cell parameters are only used when they are defined by crystal symmetry. An approximate (isotropic) treatment of cell esds is used for estimating esds involving l.s. planes.

### (1) [3-(3-Nitrophenyl)oxiran-2-yl](phenyl)methanone Fractional atomic coordinates and isotropic or equivalent isotropic displacement parameters (Å^2^)

**Table:**

	*x*	*y*	*z*	*U*_iso_*/*U*_eq_	
O1	0.25037 (8)	0.71203 (4)	0.99764 (7)	0.02695 (13)	
O2	0.10970 (8)	0.58236 (5)	0.82016 (7)	0.03095 (15)	
O3	0.36177 (10)	0.64512 (4)	0.29974 (8)	0.03355 (15)	
O4	0.32859 (13)	0.52785 (6)	0.19647 (8)	0.0453 (2)	
N1	0.32668 (10)	0.57168 (5)	0.29372 (7)	0.02557 (14)	
C1	0.21037 (11)	0.46686 (5)	0.63244 (8)	0.02330 (14)	
H1	0.1830 (17)	0.4424 (8)	0.7101 (13)	0.031 (3)*	
C2	0.22642 (12)	0.41642 (5)	0.52697 (9)	0.02570 (15)	
H2	0.2120 (17)	0.3571 (8)	0.5326 (14)	0.033 (3)*	
C3	0.26219 (11)	0.44992 (5)	0.41329 (8)	0.02357 (14)	
H3	0.2706 (18)	0.4158 (8)	0.3413 (14)	0.032 (3)*	
C4	0.28236 (9)	0.53439 (5)	0.40956 (7)	0.01988 (13)	
C5	0.26492 (10)	0.58642 (5)	0.51200 (7)	0.01960 (13)	
H5	0.2789 (16)	0.6449 (8)	0.5052 (13)	0.027 (3)*	
C6	0.22844 (9)	0.55193 (5)	0.62477 (7)	0.01941 (13)	
C7	0.21159 (10)	0.60861 (5)	0.73395 (8)	0.02193 (14)	
H7	0.1953 (17)	0.6670 (8)	0.7099 (14)	0.032 (3)*	
C8	0.29883 (10)	0.59190 (5)	0.88324 (8)	0.02108 (13)	
H8	0.3656 (16)	0.5410 (8)	0.9094 (13)	0.029 (3)*	
C9	0.35983 (10)	0.66437 (4)	0.97908 (7)	0.01953 (13)	
C10	0.55753 (10)	0.67524 (4)	1.04317 (7)	0.01900 (12)	
C11	0.62448 (11)	0.72792 (5)	1.15652 (8)	0.02299 (14)	
H11	0.5381 (18)	0.7560 (9)	1.1946 (14)	0.033 (3)*	
C12	0.80912 (12)	0.73856 (5)	1.21587 (10)	0.02919 (17)	
H12	0.856 (2)	0.7750 (10)	1.2949 (15)	0.043 (4)*	
C13	0.92704 (12)	0.69765 (6)	1.16210 (12)	0.0331 (2)	
H13	1.053 (2)	0.7034 (10)	1.2027 (17)	0.048 (4)*	
C14	0.86192 (12)	0.64582 (6)	1.04894 (12)	0.03278 (19)	
H14	0.948 (2)	0.6188 (10)	1.0128 (17)	0.052 (4)*	
C15	0.67737 (11)	0.63440 (5)	0.98993 (9)	0.02569 (15)	
H15	0.6281 (19)	0.5978 (9)	0.9086 (15)	0.039 (3)*	

### (1) [3-(3-Nitrophenyl)oxiran-2-yl](phenyl)methanone Atomic displacement parameters (Å^2^)

**Table:**

	*U*^11^	*U*^22^	*U*^33^	*U*^12^	*U*^13^	*U*^23^
O1	0.0250 (3)	0.0243 (3)	0.0327 (3)	0.0034 (2)	0.0109 (2)	−0.0014 (2)
O2	0.0229 (3)	0.0469 (4)	0.0248 (3)	−0.0096 (2)	0.0101 (2)	−0.0091 (3)
O3	0.0413 (4)	0.0311 (3)	0.0333 (3)	−0.0001 (3)	0.0191 (3)	0.0067 (3)
O4	0.0700 (6)	0.0447 (4)	0.0303 (4)	0.0012 (4)	0.0286 (4)	−0.0066 (3)
N1	0.0262 (3)	0.0311 (3)	0.0211 (3)	0.0044 (2)	0.0100 (2)	0.0018 (2)
C1	0.0259 (3)	0.0218 (3)	0.0206 (3)	−0.0013 (2)	0.0052 (3)	0.0019 (2)
C2	0.0295 (4)	0.0191 (3)	0.0257 (4)	−0.0018 (3)	0.0051 (3)	−0.0010 (3)
C3	0.0246 (3)	0.0219 (3)	0.0221 (3)	0.0010 (2)	0.0046 (3)	−0.0040 (3)
C4	0.0188 (3)	0.0226 (3)	0.0175 (3)	0.0021 (2)	0.0049 (2)	0.0002 (2)
C5	0.0207 (3)	0.0189 (3)	0.0184 (3)	0.0015 (2)	0.0053 (2)	0.0004 (2)
C6	0.0191 (3)	0.0211 (3)	0.0165 (3)	0.0017 (2)	0.0036 (2)	0.0002 (2)
C7	0.0234 (3)	0.0241 (3)	0.0180 (3)	0.0040 (2)	0.0062 (2)	0.0001 (2)
C8	0.0231 (3)	0.0205 (3)	0.0182 (3)	−0.0017 (2)	0.0048 (2)	−0.0002 (2)
C9	0.0221 (3)	0.0191 (3)	0.0178 (3)	−0.0001 (2)	0.0070 (2)	0.0009 (2)
C10	0.0213 (3)	0.0186 (3)	0.0183 (3)	−0.0012 (2)	0.0080 (2)	−0.0003 (2)
C11	0.0269 (3)	0.0198 (3)	0.0222 (3)	−0.0030 (2)	0.0079 (3)	−0.0021 (2)
C12	0.0297 (4)	0.0233 (3)	0.0294 (4)	−0.0077 (3)	0.0024 (3)	0.0009 (3)
C13	0.0221 (3)	0.0280 (4)	0.0459 (5)	−0.0042 (3)	0.0063 (3)	0.0093 (4)
C14	0.0257 (4)	0.0331 (4)	0.0447 (5)	0.0023 (3)	0.0186 (4)	0.0049 (4)
C15	0.0270 (3)	0.0270 (4)	0.0267 (4)	0.0007 (3)	0.0138 (3)	−0.0023 (3)

### (1) [3-(3-Nitrophenyl)oxiran-2-yl](phenyl)methanone Geometric parameters (Å, º)

**Table:**

O1—C9	1.2159 (9)	C7—H7	0.978 (14)
O2—C7	1.4314 (10)	C7—C8	1.4796 (11)
O2—C8	1.4225 (10)	C8—H8	0.970 (13)
O3—N1	1.2220 (10)	C8—C9	1.5067 (11)
O4—N1	1.2254 (10)	C9—C10	1.4867 (10)
N1—C4	1.4664 (10)	C10—C11	1.3978 (10)
C1—H1	0.970 (13)	C10—C15	1.3964 (10)
C1—C2	1.3905 (12)	C11—H11	0.994 (13)
C1—C6	1.3945 (11)	C11—C12	1.3888 (12)
C2—H2	0.974 (14)	C12—H12	0.972 (15)
C2—C3	1.3895 (12)	C12—C13	1.3882 (15)
C3—H3	0.939 (13)	C13—H13	0.946 (16)
C3—C4	1.3838 (11)	C13—C14	1.3866 (16)
C4—C5	1.3851 (10)	C14—H14	0.969 (16)
C5—H5	0.962 (13)	C14—C15	1.3895 (13)
C5—C6	1.3902 (10)	C15—H15	0.992 (14)
C6—C7	1.4838 (10)		
			
C8—O2—C7	62.46 (5)	O2—C8—C7	59.07 (5)
O3—N1—O4	123.16 (8)	O2—C8—H8	115.1 (7)
O3—N1—C4	118.12 (7)	O2—C8—C9	116.43 (6)
O4—N1—C4	118.72 (8)	C7—C8—H8	118.0 (7)
C2—C1—H1	119.3 (8)	C7—C8—C9	117.97 (7)
C2—C1—C6	120.32 (7)	C9—C8—H8	117.3 (7)
C6—C1—H1	120.3 (8)	O1—C9—C8	120.45 (7)
C1—C2—H2	119.8 (8)	O1—C9—C10	123.02 (7)
C3—C2—C1	120.55 (7)	C10—C9—C8	116.50 (6)
C3—C2—H2	119.7 (8)	C11—C10—C9	119.79 (7)
C2—C3—H3	120.4 (8)	C15—C10—C9	120.69 (7)
C4—C3—C2	117.86 (7)	C15—C10—C11	119.52 (7)
C4—C3—H3	121.7 (8)	C10—C11—H11	118.9 (8)
C3—C4—N1	119.41 (7)	C12—C11—C10	119.83 (8)
C3—C4—C5	123.01 (7)	C12—C11—H11	121.3 (8)
C5—C4—N1	117.58 (7)	C11—C12—H12	120.1 (9)
C4—C5—H5	120.5 (8)	C13—C12—C11	120.17 (8)
C4—C5—C6	118.39 (7)	C13—C12—H12	119.8 (9)
C6—C5—H5	121.1 (7)	C12—C13—H13	121.1 (10)
C1—C6—C7	122.54 (7)	C14—C13—C12	120.44 (8)
C5—C6—C1	119.86 (7)	C14—C13—H13	118.5 (10)
C5—C6—C7	117.60 (7)	C13—C14—H14	118.5 (10)
O2—C7—C6	118.63 (7)	C13—C14—C15	119.62 (8)
O2—C7—H7	112.9 (8)	C15—C14—H14	121.9 (10)
O2—C7—C8	58.48 (5)	C10—C15—H15	118.7 (8)
C6—C7—H7	116.7 (8)	C14—C15—C10	120.42 (8)
C8—C7—C6	122.36 (7)	C14—C15—H15	120.8 (8)
C8—C7—H7	114.6 (8)		
			
O1—C9—C10—C11	17.50 (11)	C5—C6—C7—O2	156.24 (7)
O1—C9—C10—C15	−161.44 (8)	C5—C6—C7—C8	−134.82 (8)
O2—C7—C8—C9	−105.63 (8)	C6—C1—C2—C3	0.57 (12)
O2—C8—C9—O1	−1.28 (11)	C6—C7—C8—O2	−106.07 (8)
O2—C8—C9—C10	−178.97 (6)	C6—C7—C8—C9	148.30 (7)
O3—N1—C4—C3	−172.68 (7)	C7—O2—C8—C9	108.23 (7)
O3—N1—C4—C5	6.50 (11)	C7—C8—C9—O1	66.02 (10)
O4—N1—C4—C3	6.60 (11)	C7—C8—C9—C10	−111.67 (8)
O4—N1—C4—C5	−174.22 (8)	C8—O2—C7—C6	112.36 (7)
N1—C4—C5—C6	−178.05 (6)	C8—C9—C10—C11	−164.87 (7)
C1—C2—C3—C4	0.47 (12)	C8—C9—C10—C15	16.19 (10)
C1—C6—C7—O2	−24.23 (11)	C9—C10—C11—C12	−179.56 (7)
C1—C6—C7—C8	44.72 (11)	C9—C10—C15—C14	178.98 (8)
C2—C1—C6—C5	−0.81 (11)	C10—C11—C12—C13	0.61 (13)
C2—C1—C6—C7	179.66 (7)	C11—C10—C15—C14	0.05 (12)
C2—C3—C4—N1	177.80 (7)	C11—C12—C13—C14	−0.05 (14)
C2—C3—C4—C5	−1.33 (11)	C12—C13—C14—C15	−0.52 (14)
C3—C4—C5—C6	1.10 (11)	C13—C14—C15—C10	0.52 (14)
C4—C5—C6—C1	0.00 (10)	C15—C10—C11—C12	−0.61 (12)
C4—C5—C6—C7	179.55 (6)		

### (1) [3-(3-Nitrophenyl)oxiran-2-yl](phenyl)methanone Hydrogen-bond geometry (Å, º)

**Table:**

*D*—H···*A*	*D*—H	H···*A*	*D*···*A*	*D*—H···*A*
C5—H5···O1^i^	0.962 (13)	2.335 (13)	3.2791 (11)	167.0 (11)
C13—H13···O3^ii^	0.945 (17)	2.490 (17)	3.3530 (13)	152.0 (13)
C15—H15···O4^iii^	0.992 (14)	2.381 (15)	3.2426 (13)	144.8 (12)

Symmetry codes: (i) *x*, −*y*+3/2, *z*−1/2; (ii) *x*+1, *y*, *z*+1; (iii) −*x*+1, −*y*+1, −*z*+1.

### (2) [3-(3-Nitrophenyl)oxiran-2-yl](phenyl)methanone Crystal data

**Table:**

C_15_H_11_NO_4_	*D*_x_ = 1.434 Mg m^−^^3^
*M**_r_* = 269.25	Cu *K*α radiation, λ = 1.54178 Å
Orthorhombic, *P*2_1_2_1_2_1_	Cell parameters from 9668 reflections
*a* = 4.1615 (2) Å	θ = 3.7–77.7°
*b* = 14.7498 (6) Å	µ = 0.88 mm^−^^1^
*c* = 20.3168 (8) Å	*T* = 173 K
*V* = 1247.07 (9) Å^3^	Needle, colourless
*Z* = 4	0.44 × 0.07 × 0.06 mm
*F*(000) = 560	

### (2) [3-(3-Nitrophenyl)oxiran-2-yl](phenyl)methanone Data collection

**Table:**

Bruker PHOTON-100 CMOS diffractometer	2435 reflections with *I* > 2σ(*I*)
Radiation source: sealedtube	*R*_int_ = 0.037
φ and ω scans	θ_max_ = 78.6°, θ_min_ = 3.7°
Absorption correction: multi-scan (SADABS; Bruker, 2014)	*h* = −5→5
*T*_min_ = 0.798, *T*_max_ = 0.950	*k* = −18→18
39306 measured reflections	*l* = −23→22
2640 independent reflections	

### (2) [3-(3-Nitrophenyl)oxiran-2-yl](phenyl)methanone Refinement

**Table:**

Refinement on *F*^2^	Hydrogen site location: mixed
Least-squares matrix: full	H atoms treated by a mixture of independent and constrained refinement
*R*[*F*^2^ > 2σ(*F*^2^)] = 0.029	*w* = 1/[σ^2^(*F*_o_^2^) + (0.0345*P*)^2^ + 0.1972*P*] where *P* = (*F*_o_^2^ + 2*F*_c_^2^)/3
*wR*(*F*^2^) = 0.071	(Δ/σ)_max_ = 0.001
*S* = 1.06	Δρ_max_ = 0.13 e Å^−^^3^
2640 reflections	Δρ_min_ = −0.15 e Å^−^^3^
197 parameters	Absolute structure: Refined as an inversion twin
0 restraints	Absolute structure parameter: 0.3 (2)

### (2) [3-(3-Nitrophenyl)oxiran-2-yl](phenyl)methanone Special details

**Table:**

Experimental. SADABS-2014/5 (Bruker,2014/5) was used for absorption correction. wR2(int) was 0.0614 before and 0.0562 after correction. The Ratio of minimum to maximum transmission is 0.8403. The λ/2 correction factor is 0.00150.
Geometry. All esds (except the esd in the dihedral angle between two l.s. planes) are estimated using the full covariance matrix. The cell esds are taken into account individually in the estimation of esds in distances, angles and torsion angles; correlations between esds in cell parameters are only used when they are defined by crystal symmetry. An approximate (isotropic) treatment of cell esds is used for estimating esds involving l.s. planes.
Refinement. Refined as a 2-component inversion twin.

### (2) [3-(3-Nitrophenyl)oxiran-2-yl](phenyl)methanone Fractional atomic coordinates and isotropic or equivalent isotropic displacement parameters (Å^2^)

**Table:**

	*x*	*y*	*z*	*U*_iso_*/*U*_eq_	
O1	0.6417 (4)	0.36468 (8)	0.45872 (6)	0.0372 (3)	
O2	0.9762 (3)	0.34403 (8)	0.57437 (6)	0.0356 (3)	
O3	0.2003 (5)	0.02869 (10)	0.71337 (7)	0.0606 (5)	
O4	0.2482 (5)	0.05976 (11)	0.81622 (7)	0.0567 (5)	
N1	0.2991 (5)	0.07647 (11)	0.75797 (7)	0.0395 (4)	
C1	0.8151 (5)	0.31290 (13)	0.71103 (9)	0.0335 (4)	
H1	0.934 (3)	0.3678 (12)	0.7004 (3)	0.040*	
C2	0.7899 (5)	0.28508 (14)	0.77607 (9)	0.0383 (5)	
H2	0.894 (2)	0.3214 (8)	0.8115 (8)	0.046*	
C3	0.6211 (5)	0.20742 (13)	0.79219 (10)	0.0363 (4)	
H3	0.6010 (7)	0.1885 (4)	0.8363 (10)	0.044*	
C4	0.4826 (5)	0.15840 (12)	0.74146 (8)	0.0306 (4)	
C5	0.5055 (4)	0.18379 (11)	0.67600 (8)	0.0273 (4)	
H5	0.411 (2)	0.1484 (8)	0.6426 (7)	0.033*	
C6	0.6718 (4)	0.26278 (11)	0.66071 (8)	0.0268 (4)	
C7	0.6967 (4)	0.29204 (11)	0.59069 (8)	0.0264 (3)	
H7	0.633 (5)	0.2466 (13)	0.5566 (9)	0.032*	
C8	0.6695 (4)	0.38876 (11)	0.57279 (9)	0.0272 (3)	
H8	0.635 (5)	0.4318 (13)	0.6078 (9)	0.033*	
C9	0.5571 (4)	0.41215 (12)	0.50441 (8)	0.0266 (4)	
C10	0.3543 (4)	0.49418 (11)	0.49357 (8)	0.0257 (4)	
C11	0.2260 (4)	0.54583 (11)	0.54460 (9)	0.0281 (4)	
H11	0.2629 (9)	0.5280 (4)	0.5902 (9)	0.034*	
C12	0.0454 (5)	0.62269 (12)	0.53064 (10)	0.0354 (4)	
H12	−0.050 (2)	0.6595 (8)	0.5677 (8)	0.043*	
C13	−0.0041 (5)	0.64890 (14)	0.46594 (10)	0.0411 (5)	
H13	−0.129 (3)	0.7042 (14)	0.4561 (3)	0.049*	
C14	0.1213 (5)	0.59745 (14)	0.41521 (10)	0.0425 (5)	
H14	0.0836 (10)	0.6162 (4)	0.3684 (11)	0.051*	
C15	0.2985 (5)	0.52018 (13)	0.42860 (9)	0.0340 (4)	
H15	0.3863 (19)	0.4831 (8)	0.3917 (8)	0.041*	

### (2) [3-(3-Nitrophenyl)oxiran-2-yl](phenyl)methanone Atomic displacement parameters (Å^2^)

**Table:**

	*U*^11^	*U*^22^	*U*^33^	*U*^12^	*U*^13^	*U*^23^
O1	0.0483 (8)	0.0309 (7)	0.0324 (7)	0.0018 (6)	0.0107 (6)	−0.0037 (5)
O2	0.0210 (6)	0.0352 (7)	0.0505 (8)	0.0011 (5)	0.0039 (6)	0.0093 (6)
O3	0.0981 (14)	0.0469 (9)	0.0369 (8)	−0.0287 (10)	−0.0071 (8)	0.0088 (6)
O4	0.0853 (14)	0.0539 (9)	0.0308 (8)	−0.0030 (9)	0.0051 (7)	0.0159 (6)
N1	0.0533 (11)	0.0351 (9)	0.0302 (9)	0.0034 (8)	−0.0007 (7)	0.0098 (6)
C1	0.0293 (9)	0.0336 (10)	0.0376 (10)	0.0009 (8)	−0.0035 (8)	−0.0067 (7)
C2	0.0380 (11)	0.0436 (11)	0.0333 (11)	0.0052 (9)	−0.0082 (8)	−0.0118 (8)
C3	0.0408 (11)	0.0420 (11)	0.0260 (9)	0.0123 (9)	−0.0045 (7)	−0.0017 (7)
C4	0.0354 (9)	0.0280 (9)	0.0285 (9)	0.0077 (8)	−0.0014 (7)	0.0029 (6)
C5	0.0324 (9)	0.0265 (8)	0.0231 (8)	0.0045 (8)	−0.0014 (7)	−0.0011 (6)
C6	0.0248 (9)	0.0258 (8)	0.0297 (9)	0.0047 (7)	0.0003 (7)	−0.0017 (6)
C7	0.0235 (8)	0.0248 (8)	0.0307 (9)	0.0007 (7)	0.0038 (7)	−0.0003 (6)
C8	0.0243 (8)	0.0245 (8)	0.0327 (9)	0.0005 (7)	−0.0011 (7)	−0.0014 (7)
C9	0.0261 (9)	0.0240 (9)	0.0298 (9)	−0.0046 (7)	0.0044 (7)	−0.0009 (6)
C10	0.0252 (8)	0.0236 (8)	0.0282 (9)	−0.0054 (7)	0.0014 (7)	0.0015 (6)
C11	0.0290 (9)	0.0242 (8)	0.0312 (9)	−0.0018 (7)	−0.0015 (7)	−0.0004 (6)
C12	0.0317 (10)	0.0278 (9)	0.0468 (11)	−0.0004 (8)	−0.0017 (8)	−0.0033 (8)
C13	0.0361 (10)	0.0307 (10)	0.0565 (13)	0.0032 (9)	−0.0058 (9)	0.0116 (8)
C14	0.0397 (11)	0.0480 (12)	0.0399 (11)	−0.0015 (9)	−0.0056 (9)	0.0167 (9)
C15	0.0333 (10)	0.0381 (10)	0.0304 (9)	−0.0040 (8)	0.0009 (8)	0.0042 (7)

### (2) [3-(3-Nitrophenyl)oxiran-2-yl](phenyl)methanone Geometric parameters (Å, º)

**Table:**

O1—C9	1.215 (2)	C7—H7	1.000 (19)
O2—C7	1.432 (2)	C7—C8	1.477 (2)
O2—C8	1.437 (2)	C8—H8	0.964 (19)
O3—N1	1.219 (2)	C8—C9	1.506 (2)
O4—N1	1.227 (2)	C9—C10	1.491 (2)
N1—C4	1.468 (2)	C10—C11	1.393 (2)
C1—H1	0.97 (2)	C10—C15	1.394 (2)
C1—C2	1.388 (3)	C11—H11	0.974 (19)
C1—C6	1.395 (2)	C11—C12	1.389 (2)
C2—H2	1.00 (2)	C12—H12	1.01 (2)
C2—C3	1.383 (3)	C12—C13	1.386 (3)
C3—H3	0.94 (2)	C13—H13	0.99 (2)
C3—C4	1.385 (3)	C13—C14	1.382 (3)
C4—C5	1.385 (2)	C14—H14	1.00 (2)
C5—H5	0.943 (19)	C14—C15	1.385 (3)
C5—C6	1.390 (2)	C15—H15	1.00 (2)
C6—C7	1.490 (2)		
			
C7—O2—C8	61.95 (10)	O2—C8—C7	58.85 (11)
O3—N1—O4	122.84 (18)	O2—C8—H8	114.7 (13)
O3—N1—C4	118.77 (15)	O2—C8—C9	113.67 (14)
O4—N1—C4	118.38 (16)	C7—C8—H8	117.8 (11)
C2—C1—H1	119.7	C7—C8—C9	118.19 (15)
C2—C1—C6	120.58 (18)	C9—C8—H8	118.9 (12)
C6—C1—H1	119.7	O1—C9—C8	118.87 (16)
C1—C2—H2	119.7	O1—C9—C10	121.25 (15)
C3—C2—C1	120.59 (18)	C10—C9—C8	119.85 (14)
C3—C2—H2	119.7	C11—C10—C9	123.41 (15)
C2—C3—H3	121.1	C11—C10—C15	119.37 (16)
C2—C3—C4	117.87 (18)	C15—C10—C9	117.21 (15)
C4—C3—H3	121.1	C10—C11—H11	119.9
C3—C4—N1	118.44 (16)	C12—C11—C10	120.11 (16)
C3—C4—C5	123.03 (18)	C12—C11—H11	119.9
C5—C4—N1	118.52 (16)	C11—C12—H12	119.9
C4—C5—H5	120.8	C13—C12—C11	120.10 (18)
C4—C5—C6	118.39 (16)	C13—C12—H12	119.9
C6—C5—H5	120.8	C12—C13—H13	120.1
C1—C6—C7	121.08 (16)	C14—C13—C12	119.89 (19)
C5—C6—C1	119.53 (16)	C14—C13—H13	120.1
C5—C6—C7	119.39 (15)	C13—C14—H14	119.8
O2—C7—C6	115.65 (15)	C13—C14—C15	120.43 (18)
O2—C7—H7	114.4 (12)	C15—C14—H14	119.8
O2—C7—C8	59.20 (10)	C10—C15—H15	120.0
C6—C7—H7	116.7 (11)	C14—C15—C10	120.09 (18)
C8—C7—C6	120.63 (15)	C14—C15—H15	120.0
C8—C7—H7	117.2 (11)		
			
O1—C9—C10—C11	173.70 (17)	C5—C6—C7—O2	−151.82 (15)
O1—C9—C10—C15	−7.5 (2)	C5—C6—C7—C8	140.25 (17)
O2—C7—C8—C9	102.02 (17)	C6—C1—C2—C3	−0.3 (3)
O2—C8—C9—O1	29.4 (2)	C6—C7—C8—O2	103.45 (18)
O2—C8—C9—C10	−148.64 (14)	C6—C7—C8—C9	−154.53 (16)
O3—N1—C4—C3	−173.69 (19)	C7—O2—C8—C9	−109.74 (16)
O3—N1—C4—C5	7.2 (3)	C7—C8—C9—O1	−36.7 (2)
O4—N1—C4—C3	7.3 (3)	C7—C8—C9—C10	145.30 (17)
O4—N1—C4—C5	−171.84 (18)	C8—O2—C7—C6	−111.82 (17)
N1—C4—C5—C6	178.22 (17)	C8—C9—C10—C11	−8.3 (2)
C1—C2—C3—C4	0.7 (3)	C8—C9—C10—C15	170.47 (16)
C1—C6—C7—O2	27.6 (2)	C9—C10—C11—C12	178.49 (17)
C1—C6—C7—C8	−40.4 (3)	C9—C10—C15—C14	−177.84 (17)
C2—C1—C6—C5	−0.7 (3)	C10—C11—C12—C13	−0.9 (3)
C2—C1—C6—C7	179.91 (18)	C11—C10—C15—C14	1.0 (3)
C2—C3—C4—N1	−179.20 (17)	C11—C12—C13—C14	1.3 (3)
C2—C3—C4—C5	−0.1 (3)	C12—C13—C14—C15	−0.6 (3)
C3—C4—C5—C6	−0.8 (3)	C13—C14—C15—C10	−0.6 (3)
C4—C5—C6—C1	1.2 (3)	C15—C10—C11—C12	−0.3 (3)
C4—C5—C6—C7	−179.36 (16)		

### (2) [3-(3-Nitrophenyl)oxiran-2-yl](phenyl)methanone Hydrogen-bond geometry (Å, º)

**Table:**

*D*—H···*A*	*D*—H	H···*A*	*D*···*A*	*D*—H···*A*
C5—H5···O1^i^	0.94 (1)	2.35 (1)	3.209 (2)	151 (1)
C8—H8···O4^ii^	0.96 (2)	2.49 (2)	3.401 (2)	158 (2)
C15—H15···O3^iii^	1.00 (1)	2.51 (2)	3.411 (2)	150 (1)

Symmetry codes: (i) *x*−1/2, −*y*+1/2, −*z*+1; (ii) −*x*+1, *y*+1/2, −*z*+3/2; (iii) *x*+1/2, −*y*+1/2, −*z*+1.
